# *Wolbachia pipientis* Associated With Tephritid Fruit Fly Pests: From Basic Research to Applications

**DOI:** 10.3389/fmicb.2020.01080

**Published:** 2020-06-03

**Authors:** Mariana Mateos, Humberto Martinez Montoya, Silvia B. Lanzavecchia, Claudia Conte, Karina Guillén, Brenda M. Morán-Aceves, Jorge Toledo, Pablo Liedo, Elias D. Asimakis, Vangelis Doudoumis, Georgios A. Kyritsis, Nikos T. Papadopoulos, Antonios A. Augustinos, Diego F. Segura, George Tsiamis

**Affiliations:** ^1^Departments of Ecology and Conservation Biology, and Wildlife and Fisheries Sciences, Texas A&M University, College Station, TX, United States; ^2^Laboratorio de Genética y Genómica Comparativa, Unidad Académica Multidisciplinaria Reynosa Aztlan, Universidad Autónoma de Tamaulipas, Ciudad Victoria, Mexico; ^3^Instituto de Genética ‘Ewald A. Favret’ – GV IABIMO (INTA-CONICET) Hurlingham, Buenos Aires, Argentina; ^4^El Colegio de la Frontera Sur, Tapachula, Mexico; ^5^Department of Environmental Engineering, University of Patras, Agrinio, Greece; ^6^Laboratory of Entomology and Agricultural Zoology, Department of Agriculture Crop Production and Rural Environment, University of Thessaly, Larissa, Greece; ^7^Department of Plant Protection, Institute of Industrial and Forage Crops, Hellenic Agricultural Organization – DEMETER, Patras, Greece

**Keywords:** insect control, symbiosis, endosymbiont, incompatible insect technique, cytoplasmic incompatibility

## Abstract

Members of the true fruit flies (family Tephritidae) are among the most serious agricultural pests worldwide, whose control and management demands large and costly international efforts. The need for cost-effective and environmentally friendly integrated pest management (IPM) has led to the development and implementation of autocidal control strategies. These approaches include the widely used sterile insect technique and the incompatible insect technique (IIT). IIT relies on maternally transmitted bacteria (namely *Wolbachia*) to cause a conditional sterility in crosses between released mass-reared *Wolbachia*-infected males and wild females, which are either uninfected or infected with a different *Wolbachia* strain (i.e., cytoplasmic incompatibility; CI). Herein, we review the current state of knowledge on *Wolbachia*-tephritid interactions including infection prevalence in wild populations, phenotypic consequences, and their impact on life history traits. Numerous pest tephritid species are reported to harbor *Wolbachia* infections, with a subset exhibiting high prevalence. The phenotypic effects of *Wolbachia* have been assessed in very few tephritid species, due in part to the difficulty of manipulating *Wolbachia* infection (removal or transinfection). Based on recent methodological advances (high-throughput DNA sequencing) and breakthroughs concerning the mechanistic basis of CI, we suggest research avenues that could accelerate generation of necessary knowledge for the potential use of *Wolbachia*-based IIT in area-wide integrated pest management (AW-IPM) strategies for the population control of tephritid pests.

## Introduction

### The Economic Importance and Management of Tephritid Pest Species

Flies in the family Tephritidae (Diptera) include some of the world’s most important agricultural pests. The family is comprised of ∼4,900 described species within 481 genera, of which six (*Anastrepha*, *Bactrocera*, *Ceratitis*, *Dacus*, *Rhagoletis*, and *Zeugodacus*) contain ∼70 major pest species (White and Elson-Harris, 1992; Norrbom, 2004a, b, 2010; Mengual et al., 2017). Pest tephritids represent an enormous economic cost because they cause direct losses to a diversity of crops (fruits, vegetables, and flowers) (White and Elson-Harris, 1992). Furthermore, they hamper the development of agriculture in numerous countries, due to the strict quarantines imposed by countries importing affected crops, and to the huge costs associated with efforts aimed at prevention, containment, suppression, and eradication.

To prevent or minimize the harmful effects of tephritid pests, growers must comply with health and safety standards required by the market, applying an area-wide management approach involving chemical, biological, cultural, and autocidal control practices (Reyes et al., 2000; Enkerlin, 2005). Autocidal refers to methods that use the insect to control itself, by releasing insects that are sterile or induce sterility upon mating with wild insects in the next or subsequent generations (Black et al., 2011; Leftwich et al., 2014; Handler, 2016). Autocidal strategies include the sterile insect technique (SIT) (Knipling, 1955; Hendrichs and Robinson, 2009); one of the most widespread control methods used against fruit flies (reviewed in Dias et al., 2018). SIT relies on the mass-rearing production, sterilization and recurrent release of insects (preferentially males) of the targeted species. Sterilization is typically attained by radiation (Bakri et al., 2005), in a way that does not impair male mating and insemination capabilities. Wild females that mate with sterilized males lay unfertilized eggs. At the appropriate sterile:wild (S:W) ratio, the reproductive potential of the target population can be reduced (Knipling, 1955; Klassen and Curtis, 2005; Cáceres et al., 2007). Historically, at least 28 countries have used the SIT at a large-scale for the suppression or eradication of pests (Hendrichs et al., 1995, 2005; Suckling et al., 2016). SIT has been applied successfully for several non-tephritid insect pests including the New World screw worm *Cochliomyia hominivorax* (Coquerel), several species of tsetse fly (*Glossina* spp.), the codling moth *Cydia pomonella* (L.) (reviewed in Robinson, 2002b; Dyck et al., 2005; Bourtzis and Robinson, 2006), and mosquitoes (Benedict and Robinson, 2003; Lees et al., 2015). Successful SIT programs as part of Area-wide Integrated Pest Management (AW-IPM) strategies have also been implemented for several tephritids: *Ceratitis capitata* (Wiedemann); *Anastrepha ludens* (Loew); *Anastrepha obliqua* (Macquart); *Zeugodacus cucurbitae* (Coquillett); *Bactrocera dorsalis* Hendel; and *Bactrocera tryoni* (Froggatt) (Enkerlin, 2005; Hendrichs et al., 2005; Klassen and Curtis, 2005; Cáceres et al., 2007). SIT is currently being developed for three additional tephritid species: *Anastrepha fraterculus* (Wiedemann) (Cladera et al., 2014); *Dacus ciliatus* Loew (Rempoulakis et al., 2015) and *Bactrocera tau* (Walker) (Du et al., 2016). The advantages of the SIT over other pest control approaches (e.g., use of pesticides) are that it is species-specific and environmentally friendly (Lees et al., 2015; Bourtzis et al., 2016), and resistance is less likely to evolve (but see Hibino and Iwahashi, 1991; McInnis et al., 1996).

Another autocidal strategy where mating between mass-reared and wild insects can be used to suppress pest populations is the incompatible insect technique (IIT); coined by Boller et al. (1976). The earliest successful pilot application of IIT was in *Culex* mosquitoes (Laven, 1967), and interest in applying it to mosquitoes has resurged in recent years (reviewed in Ross et al., 2019b). IIT also relies on the principle of reducing female fertility, but utilizes endosymbiotic bacteria instead of radiation, to induce a context-dependent sterility in wild females. It is based on the ability of certain maternally inherited bacteria (mainly from the genus *Wolbachia*) to induce a form of reproductive incompatibility known as cytoplasmic incompatibility (CI; explained in the section below). Herein we review the current knowledge on taxonomic diversity of *Wolbachia*-tephritid associations and their phenotypic consequences, and identify gaps in knowledge and approaches in the context of potential application of IIT, alone or in combination with SIT, in AW-IPM programs to control tephritid pests.

### The Influence of *Wolbachia* on Host Ecology

Insects and other arthropods are common hosts of maternally inherited bacteria (reviewed in Duron and Hurst, 2013). These heritable endosymbionts can have a strong influence on host ecology. Typically, such vertically transmitted bacteria are vastly (or fully) dependent on the host for survival and transmission. Certain associations are obligate for both partners, and generally involve a nutritional benefit to the host. Other heritable bacteria are facultative, with such associations ranging from mutualistic to parasitic from the host’s perspective. Among these, *Wolbachia* is the most common and widespread facultative symbiont of insects and arthropods (Hilgenboecker et al., 2008; Zug and Hammerstein, 2012; de Oliveira et al., 2015; Weinert et al., 2015).

*Wolbachia* is a diverse and old genus (possibly older than 200 million years; Gerth and Bleidorn, 2016) of intracellular Gram-negative Alphaproteobacteria (within the order Rickettsiales) associated with arthropods and filarial nematodes. *Wolbachia* cells resemble small spheres 0.2–1.5 μm, occur in all tissue types, but tend to be more prevalent in ovaries and testes of infected hosts, and are closely associated with the female germline (reviewed by Harris et al., 2010; see also Sacchi et al., 2010). *Wolbachia* is estimated to infect 40–66% of insect species (Hilgenboecker et al., 2008; Zug and Hammerstein, 2012; de Oliveira et al., 2015; Weinert et al., 2015). Within a species or population, the infection prevalence of *Wolbachia* can be quite variable over space (e.g., Kriesner et al., 2016) and time (e.g., Turelli and Hoffmann, 1991, 1995).

**FIGURE 1:**
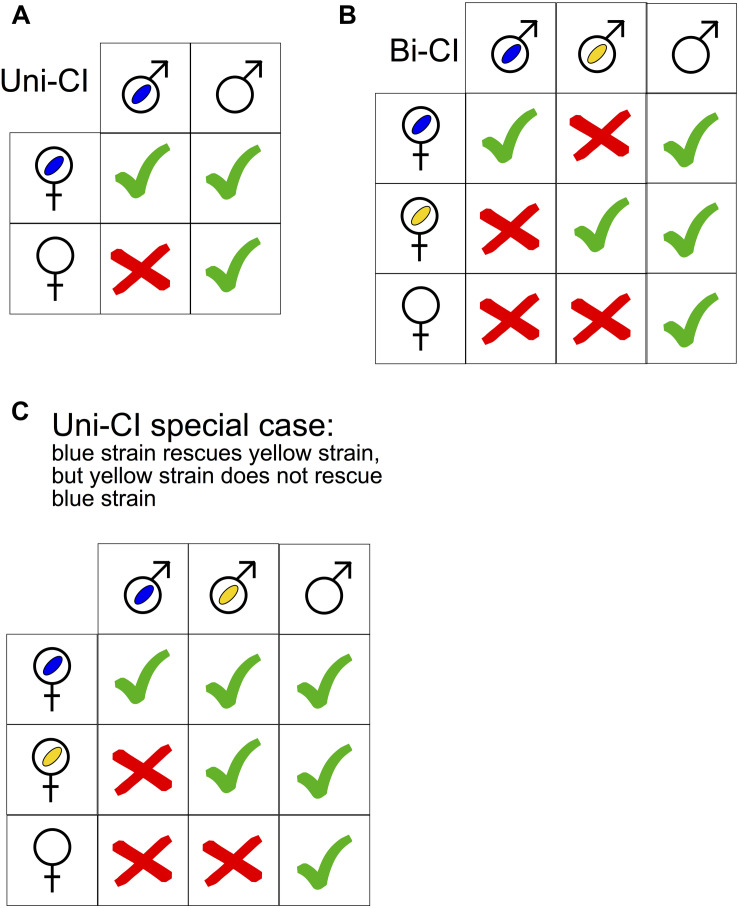
**(A,B)** Qualitative illustration of uni- and bidirectional cytoplasmic incompatibility (CI) on the basis of the *Wolbachia* infection status of the parent generation. Empty male and female symbols signify absence of *Wolbachia*. Blue and yellow ovals represent distinct *Wolbachia* strains. Green tick marks = Successful offspring production. Red crosses = no offspring production. **(C)** A special case of unidirectional incompatibility in which one *Wolbachia* strain (see text) can rescue another strain (i.e., the yellow one), but not vice versa.

The most commonly documented effects of *Wolbachia* on arthropod hosts fall under the category of reproductive parasitism, which involves manipulation of host reproduction to enhance symbiont transmission and persistence, in general by increasing the relative frequency of *Wolbachia*-infected vs. uninfected females. Females are typically the sex that can transmit *Wolbachia* and other heritable bacteria, although rare exceptions exist (Hoffmann and Turelli, 1988; Moran and Dunbar, 2006; Chafee et al., 2010). *Wolbachia* employs all four types of reproductive manipulation (reviewed by Werren et al., 2008; Saridaki and Bourtzis, 2010; Schneider et al., 2011). Feminization converts genetic males into functional females, and occurs in the orders Hemiptera, Lepidoptera, and Isopoda. *Wolbachia*-induced parthenogenesis occurs in haplo-diploid hosts (e.g., Acari, Hymenoptera, and Thysanoptera), where unfertilized eggs, which would otherwise develop into males, develop into females. Male killing causes death of infected males to the presumed advantage of surviving infected female siblings, and occurs in Coleoptera, Diptera, Lepidoptera, and Pseudoscorpionida. Cytoplasmic incompatibility (CI) (Yen and Barr, 1971) prevents infected males from producing viable offspring upon mating with females lacking *Wolbachia* (or a compatible strain of *Wolbachia*; see below; Figure 1). CI is the most commonly reported *Wolbachia*-induced reproductive phenotype, and is found in Acari, Coleoptera, Diptera, Hemiptera, Hymenoptera, Isopoda, Lepidoptera, and Orthoptera.

Cytoplasmic incompatibility was discovered almost half a century ago (Yen and Barr, 1971), but its mechanism has not been fully elucidated. A useful conceptual model to understand the observed patterns of CI is “mod/resc” (Hurst, 1991; Werren, 1997). It postulates that *Wolbachia* has two functions: *mod* (for modification), which acts as a toxin or imprint of the male germline; and *resc* (for rescue), which acts as an antidote. The *mod* function acts on the nucleus in the male germline, before *Wolbachia* are shed from maturing sperm (Presgraves, 2000). When a sperm nucleus affected by *mod* enters the egg of an uninfected female, this nucleus encounters problems such as delays in DNA replication and cell-cycle progression, leading to embryo death. In contrast, if the appropriate *resc* (“the antidote”) function is active in the egg, the defect caused by *mod* in the sperm is rescued, and the embryo proceeds through normal development. In the case of unidirectional CI (uni-CI), all or some of the eggs from uninfected females that are fertilized by sperm from *Wolbachia*-infected males (the “CI cross”) fail to develop (Figure 1A). *Wolbachia*-infected females are compatible with uninfected males, and thus have a reproductive advantage over their *Wolbachia*-uninfected counterparts. Consequently, above a certain threshold of *Wolbachia* infection frequency in a host population, *Wolbachia* frequencies can rapidly increase to a stable equilibrium frequency. When CI is strong (e.g., all embryos from the CI cross fail), fitness costs of *Wolbachia* are low, and maternal (vertical) transmission is high, the threshold *Wolbachia* frequency to achieve invasion can be close to zero, and the stable equilibrium frequency can be close to 100% (Caspari and Watson, 1959; Turelli and Hoffmann, 1999; Rasgon, 2008). Bi-directional CI (bi-CI) results from crosses involving two different (incompatible) *Wolbachia* strains (Figure 1B). Crosses between females and males infected with the same or compatible *Wolbachia* strains are viable. Under bi-CI between two *Wolbachia* strains with equivalent fitness effects on a host, the infection frequency of an introduced strain must exceed 50% to achieve invasion (Rousset et al., 1991; Dobson et al., 2002). Special cases of uni-CI and bi-CI patterns can occur. For example, a strain may not induce CI, but is able to rescue the defect caused by a different strain (Figure 1C) (Zabalou et al., 2004a).

Several recent breakthrough studies have collectively identified *Wolbachia*-encoded genes (of viral origin) that contribute to the induction and rescue of CI (Beckmann et al., 2017; LePage et al., 2017; Bonneau et al., 2018, 2019; Lindsey et al., 2018; Shropshire et al., 2018; Beckmann et al., 2019c; Chen et al., 2019; Shropshire and Bordenstein, 2019). *Wolbachia*-encoded genes that rescue CI are labeled as *cifA*, *cidA*, or *cindA*, depending on whether they rescue a defect caused by deubiquitylase (d), nuclease (n), both (nd); “f” is used by certain authors and/or when the nature of the defect is unknown (see Beckmann et al., 2019a, b; Shropshire et al., 2019). In CI-inducing *Wolbachia* strains, each of the above genes occurs upstream of a gene (its “cognate”) similarly labeled, but with a “B” replacing the “A” (i.e., *cifB*, *cidB*, or *cindB*, respectively) that seems to function as a toxin. Certain *Wolbachia* strains have more than one “A–B” pair, and the combination of these is consistent with patterns of incompatibility in *Drosophila* (LePage et al., 2017) and *Culex* (Bonneau et al., 2018). Knowledge accrued to date indicates that more than one *Wolbachia*-encoded mechanism of CI exists, and thus, information on the genes encoded by *Wolbachia* genomes can help predict expected patterns of incompatibility among strains that have not been experimentally characterized.

In addition to its reproductive phenotypes on arthropods, *Wolbachia* engages in obligate mutualistic interactions with filarial nematodes (Werren et al., 2008) and with members of five insect orders (reviewed in Zug and Hammerstein, 2015). As a facultative symbiont, *Wolbachia* can provide direct fitness benefits to its insect hosts by influencing development, nutrition, iron metabolism, lifespan, and fecundity (Dean, 2006; Aleksandrov et al., 2007; Weeks et al., 2007; Brownlie et al., 2009; Ikeya et al., 2009; Kremer et al., 2009; Newton and Rice, 2020), and most notably, by conferring resistance or tolerance to pathogens, particularly single-stranded RNA viruses (Hedges et al., 2008; Teixeira et al., 2008; Moreira et al., 2009). The interference of *Wolbachia* with the replication and transmission of certain viruses, along with its ability to spread in populations via CI, form the basis of several population replacement programs (reviewed in Ross et al., 2019b; Chrostek et al., 2020). *Wolbachia* in *Drosophila* appears to confer an additional fitness benefit in the form of increased recombination (Bryant and Newton, 2019; Singh, 2019).

Certain host-*Wolbachia* combinations incur fitness costs to the host, beyond reproductive parasitism, including reduced longevity, sperm competitive ability, and fecundity, as well as higher susceptibility to natural enemies (Hoffmann et al., 1990; Min and Benzer, 1997; Snook et al., 2000; Champion de Crespigny and Wedell, 2006; Fytrou et al., 2006; Vasquez et al., 2011; Suh et al., 2017; Sumida et al., 2017). Similarly, certain host-*Wolbachia* combinations may potentially enhance pathogen-vectoring capacities (Hughes et al., 2012b; Baton et al., 2013; Dodson et al., 2014; Murdock et al., 2014). *Wolbachia* has been reported to influence positively or negatively numerous aspects of their host’s behavior including sleep, learning and memory capacity, mating, feeding, thermal preference, locomotion, and agression (reviewed by Bi and Wang, 2019; Wedell, 2019).

## Methods to Study *Wolbachia*

### Methods to Assess *Wolbachia* Infection Status

**FIGURE 2:**
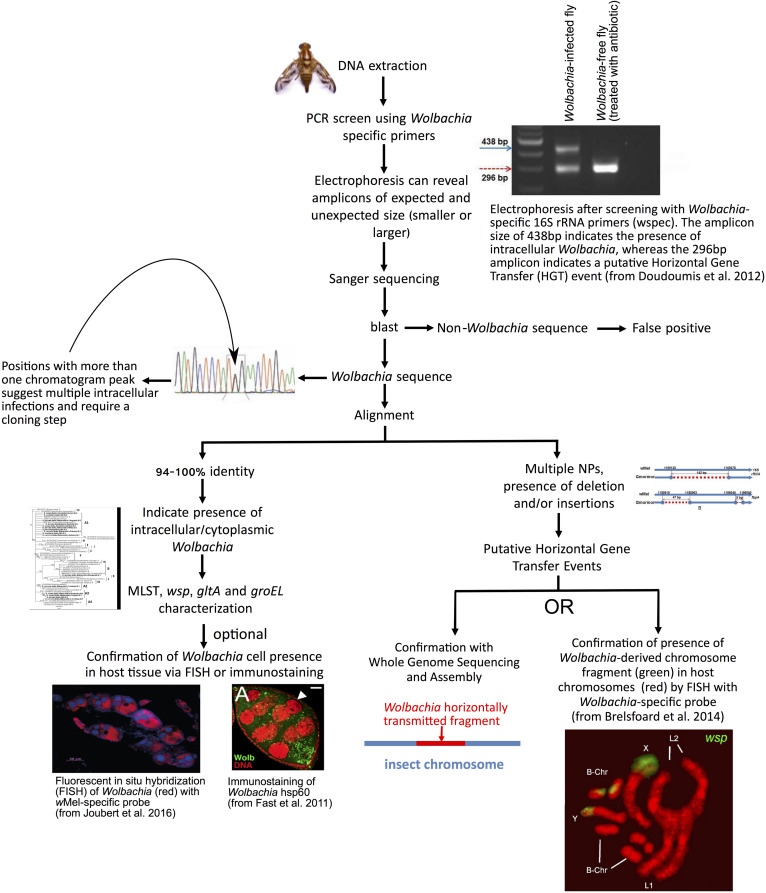
Recommended steps to screen for *Wolbachia* infections in tephritids and other arthropods. A PCR is performed with *Wolbachia*-specific primers on DNA isolated from whole, or parts of (e.g., abdomens), insect. Agarose gel electrophoresis of the PCR products is used to determine whether the amplicon is of the expected size. Amplicons of expected size are directly sequenced (e.g., Sanger method). High sequence identity to other *Wolbachia* suggests *Wolbachia* infection. Clean chromatograms are consistent with a single *Wolbachia* strain. Otherwise, a cloning step to identify different *Wolbachia* alleles is required. Other genes are then amplified and sequenced for further genetic characterization of the strain. As an optional step, localization of *Wolbachia* cells within host tissues can be achieved by Fluorescent *In Situ* Hybridization (FISH) with *Wolbachia*-specific rRNA probe or immunolabeling with antibody specific to *Wolbachia* protein. An amplicon of an unexpected size might indicate the occurrence of a horizontally transmitted *Wolbachia* genome fragment to the insect chromosome, rather than a current infection. Similarly, multiple nucleotide polymorphisms (NP) or insertions/deletions, compared to known strains, are suggestive of *Wolbachia* pseudogenes (e.g., horizontally transferred to host genome). This can be further tested by *in situ* hybridization of *Wolbachia*-specific probe to host chromosomes, and/or by Whole Genome Sequencing of host. Photo of fly (*Anastrepha obliqua*) by Fabiola Roque (ECOSUR-UT). Image from Fast et al. (2011) freely available at https://www.ncbi.nlm.nih.gov/pmc/articles/PMC4030408/bin/NIHMS391830-supplement-Supporting_Online_Material.pdf (accessed April 01, 2019). Original sources of other photographs are available in open access journals (Doudoumis et al., 2012; Brelsfoard et al., 2014; Joubert et al., 2016).

For purposes of this review, we consider a host species or population as “infected” with *Wolbachia*, even if the infection is transient or found at low titer. *Wolbachia*, and most cytoplasmically transmitted endosymbionts, are fastidious to culture outside host cells, such that their study typically relies on culture-independent methods. A recommended flow-chart of steps is depicted in Figure 2. The most utilized approach to date for identifying hosts infected with *Wolbachia* is through PCR screening of *Wolbachia* genes in DNA extracts of hosts. Different PCR primers have been used to perform such surveys, traditionally targeting a portion of the *16S ribosomal (r)RN*A gene or of a ubiquitous protein-coding gene (e.g., *wsp* or *ftsZ*). Simoes et al. (2011) evaluated the relative sensitivity and specificity of different primer pairs aimed at *Wolbachia* detection and identification, revealing that no single PCR protocol is capable of specific detection of all known *Wolbachia* strains. A related method known as “loop mediated isothermal amplification” (LAMP; not shown in Figure 2), which requires less infrastructure than PCR, has been successfully employed for *Wolbachia* detection in several insects (da Silva Gonçalves et al., 2014).

The two major shortcomings of utilizing solely PCR (or LAMP) to detect *Wolbachia* presence are the occurrence of false negatives and false positives. A false negative occurs when a specimen is infected by *Wolbachia*, yet the screening approach fails to detect its presence. The efficiency of the PCR can be affected by the presence of inhibitors (Marcon et al., 2011; Beckmann and Fallon, 2012), by low concentration/poor quality of the target DNA molecule, as well as type and concentration of the polymerase and other PCR reagents. At the very least, negative *Wolbachia* detection PCRs should be validated by evaluating the quality of the DNA extract, through positive amplification of a host-encoded gene (e.g., the mitochondrial Cytochrome Oxidase subunit I or single-copy nuclear genes). Several higher sensitivity approaches have been devised, particularly for low-titer infections, such as: long PCR (Jeyaprakash and Hoy, 2000); nested PCR (Arthofer et al., 2009a); quantitative PCR (Mee et al., 2015); or the design of alternative and/or more specific primers, including the use of *Wolbachia* multi-copy genes as PCR targets (Schneider et al., 2014). These methods, however, have not been widely implemented, likely due to the higher effort or cost involved.

False positives occur when a specimen not harboring *Wolbachia* is identified as *Wolbachia*-infected. Several instances have been reported where insect chromosomes carry *Wolbachia*-derived fragments, presumably from a horizontal gene transfer event that occurred at some point in the host lineage as the result of an active infection that was subsequently lost. The size of the horizontally transmitted fragment can range from ca. 500 bp to the equivalent of an entire *Wolbachia* chromosome (Dunning Hotopp et al., 2007). In some cases, entire *Wolbachia* chromosomes have been transferred more than once onto the same host genome (Brelsfoard et al., 2014; International Glossina Genome Initiative, 2014). The range of hosts carrying *Wolbachia*-derived genome fragments is broad and includes several dipterans (tephritids, *Glossina morsitans* Westwood; *Drosophila* spp., mosquitoes), other insects, as well as nematodes (Fenn et al., 2006; Dunning Hotopp et al., 2007; Nikoh et al., 2008; Brelsfoard et al., 2014; Morrow et al., 2015; Attardo et al., 2019). It is therefore desirable to corroborate PCR-based inferences with approaches that detect *Wolbachia* cells in host tissues. Such microscopy approaches can be based on nucleic acid hybridization (e.g., Chen et al., 2005) or antibody-based detection of *Wolbachia* proteins (e.g., *wsp*; Veneti et al., 2003; and *ftsZ;* Newton et al., 2015). A major drawback of these methods is that they require substantial investment in time and equipment compared to PCR-based approaches. False positives can also occur if the primers targeted at *Wolbachia* turn out to amplify a fragment of the genome of the host (not derived from *Wolbachia*) or of another symbiont of the host. Such false positives are relatively easy to rule out upon sequencing and analysis of the amplified product. Finally, as with any PCR work, false positives can result from contamination of the specimen, the DNA template, or the PCR reagents. Thus, it is important to implement adequate sterile practices and negative controls.

The above approaches require destruction of specimens for DNA isolation or for tissue fixation. As a rapid and non-destructive alternative, Near-Infrared Spectroscopy (NIR) has been developed for identification of specimens infected with *Wolbachia*, including the distinction of two different *Wolbachia* strains (Sikulu-Lord et al., 2016). This method, however, requires standardization according to species, sex, age, or any other condition that may affect absorbance, and is not 100% efficient. To our knowledge, this method has not been employed to assess *Wolbachia* infection in tephritids.

### Methods to Taxonomically Characterize *Wolbachia* Strains

The main evolutionary lineages of *Wolbachia* are assigned to “supergroups” (Zhou et al., 1998). Sixteen supergroups have been recognized to date (Glowska et al., 2015; Bleidorn and Gerth, 2017). Supergroups A and B are widespread in arthropods and are common reproductive manipulators (Baldo et al., 2006; Werren et al., 2008). Supergroups C and D are obligate mutualists of filarial nematodes, whereas supergroup F is found in both arthropods and nematodes (Ros et al., 2009). Other supergroups have more restricted host distributions (Augustinos et al., 2011). *Wolbachia* are generally compared and classified on the basis of Multilocus Sequence Typing (MLST) systems (Baldo et al., 2006; Paraskevopoulos et al., 2006). The most commonly used MLST is based on the PCR amplification of fragments of five ubiquitous genes: *cox*A, *fbp*A, *fts*Z, *hcp*A, and *ga*tB. However, this MLST system has limitations, in that not all genes are readily amplified in all *Wolbachia* strains, and it fails to distinguish among very closely related strains (Augustinos et al., 2011; Bleidorn and Gerth, 2017). Several additional genes commonly amplified and reported are the 16S *rRNA*, *gro*EL, *glt*A, and the *wolbachia surface protein* (*wsp*) (O’Neill et al., 1992; Braig et al., 1998; Zhou et al., 1998; Augustinos et al., 2011). The *wsp* gene is highly variable and shows evidence of intragenic recombination (Werren and Bartos, 2001; Ros et al., 2012). An MLST database^1^ is available to compare sequences of alleles for the five MLST loci and the *wsp* gene. Upon submission to the MLST database, new alleles for the *wsp* and for each of the MLST loci are assigned a unique number. A *Wolbachia* sequence type (ST) is defined on the basis of MLST allele combinations, with each allele combination assigned a unique ST number. Further characterization of each MLST-defined strain can be achieved by examination of four hypervariable regions (HVRs) of the *wsp* gene (Baldo et al., 2005).

Hosts can be infected by one or more distinct strains of *Wolbachia*. Traditionally, direct Sanger sequencing of PCR products that resulted in sequences with ambiguous base pairs would be subjected to cloning followed by sequencing. The allele intersection analysis method (AIA; Arthofer et al., 2011) can then be used to assign MLST alleles to *Wolbachia* strains, but it requires a priori knowledge on the number of strains present. AIA identifies pairs of multiply infected individuals that share *Wolbachia* and differ by only one strain. Alternative approaches to circumvent cloning include the use of strain-specific primers (e.g., for the *wsp* gene; Zhou et al., 1998; Arthofer et al., 2009b), or of high throughput sequencing approaches (e.g., Illumina HiSeq, MiSeq, and NovaSeq) to sequence MLST or other marker PCR amplicons (e.g., Gibson et al., 2014; Brandon-Mong et al., 2015). Primer bias, however, where the fragment of one *Wolbachia* strain is preferentially amplified over the other, has been reported (Arthofer et al., 2011), such that presence of certain *Wolbachia* strains might be missed.

Use of the MLST system alone has two major drawbacks. First, strains of *Wolbachia* sharing identical MLST or *wsp* alleles can differ from each other at other loci (Paraskevopoulos et al., 2006; Riegler et al., 2012). Secondly, the MLST, *16S rRNA*, and *wsp* loci contain limited phylogenetic signal for inferring relationships within *Wolbachia* supergroups (Bleidorn and Gerth, 2017). Therefore, to assess such intra-ST variation and to infer evolutionary relationships among closely related *Wolbachia* strains, additional (more variable) loci must be evaluated. The multiple locus variable number tandem repeat analysis developed by Riegler et al. (2012) allows distinction of closely related *Wolbachia* strains based on PCR and gel electrophoresis.

Whole genome sequencing represents a powerful approach to distinguish closely related *Wolbachia* strains, infer their evolutionary relationships, test for recombination, and identify genes of interest (e.g., Klasson et al., 2009; LePage et al., 2017). Due to its fastidious nature (but see Uribe-Alvarez et al., 2018 for a recent breakthrough) and occurrence of repetitive elements, genome sequencing and assembly of *Wolbachia* (and other host-associated uncultivable bacteria) has proven difficult. Recent advances, particularly those based on targeted hybrid enrichment (Lemmon and Lemmon, 2013) prior to high-throughput sequencing (Bleidorn, 2016; Goodwin et al., 2016) have been successfully applied to *Wolbachia* for short-read technologies (i.e., Illumina; Kent et al., 2011; Geniez et al., 2012). The combination of targeted hybrid capture and long-read technologies, such as Pacific Biosciences’ Single Molecule Real-Time (e.g., Wang et al., 2015) or Oxford Nanopore Technologies’ platforms is expected to greatly advance *Wolbachia* genomics research.

### Methods to Functionally Characterize *Wolbachia* Strains

A major challenge to investigating the effects of *Wolbachia* on a host is to generate *Wolbachia*-present and *Wolbachia*-free treatments while controlling for host genetic background. The challenge stems from the difficulty of adding or removing *Wolbachia* to/from particular hosts. Addition of *Wolbachia* to a particular host background can be achieved by transinfection (reviewed in Hughes and Rasgon, 2014). Because the vertical transmission of *Wolbachia* appears to be dependent on its close association to the host germline, successful artificial transfer of *Wolbachia* typically relies on injection of cytoplasm from a donor egg (but see Frydman et al., 2006) or early embryo into a recipient embryo via microinjection (reviewed in Hughes and Rasgon, 2014). The success rate of the transinfection procedure is generally very low; in tephritids it is 0–0.09% (calculated as the proportion of injected embryos that emerged as *Wolbachia*-infected adult females that transmitted *Wolbachia* to offspring) (Zabalou et al., 2004b, 2009; Apostolaki et al., 2011; Martinez et al., 2016). The low success rate is generally a result of the low survival of injected embryos, the low proportion of *Wolbachia*-positive survivors, and the low/incomplete transmission of *Wolbachia* to their offspring.

**FIGURE 3:**
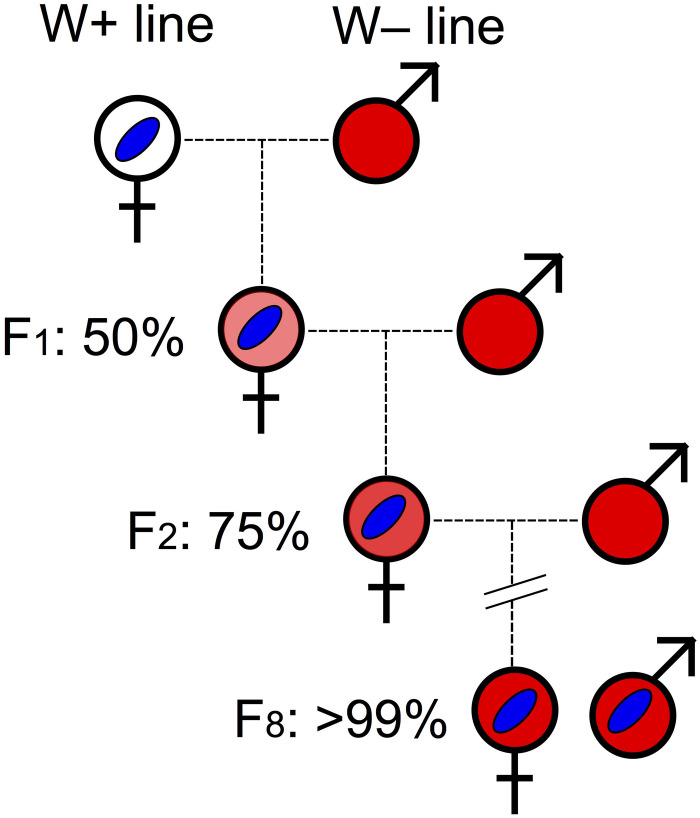
Backcrossing procedure. *Wolbachia* infection is indicated by blue oval. Host nuclear backgrounds are indicated by colors: white represents the initial nuclear background of *Wolbachia*-infected host; red (darkest) indicates the host background of the *Wolbachia*-free line contributing males every generation. Different shades of red represent the increasing replacement of “white” nuclear background over backcrossing generations (*F*_1_ to *F*_8_) by “red” nuclear background.

Intra-species (or between sibling species) transfer of *Wolbachia* to a particular host nuclear background can also be achieved through introgression, whereby males of the desired background are repeatedly backcrossed with *Wolbachia*-infected females (e.g., Dobson et al., 1999; Jaenike, 2007). Under this scheme, after eight generations of consistent backcrossing, ∼99.6% of the host nuclear background is expected to have been replaced (Figure 3). The drawback of this approach is that the mitochondrial genome will not be replaced. Therefore, the effects of mitochondrial type and *Wolbachia* infection cannot be separated.

Due to less than perfect transmission, passive loss of *Wolbachia* in certain host individuals may be used to obtain *Wolbachia*-free and *Wolbachia*-infected hosts of equivalent genetic background. *Wolbachia* removal has also been achieved by “extreme” temperature treatment (e.g., 30°C; Ribeiro, 2009). The most common way of removing *Wolbachia*, however, is achieved via antibiotic treatment, but several potential biases must be addressed (reviewed by Li et al., 2014). Antibiotic treatment is likely to alter the microbiota, other than *Wolbachia*, associated with the host. In addition, antibiotics may affect the host in a microbe-independent manner. For instance, antibiotic treatment can affect host mitochondria (Ballard and Melvin, 2007), which in turn can reduce host fitness. A common practice to circumvent these problems is to wait several generations after antibiotic treatment, and to promote “restoration” of the host’s pre-antibiotic microbiota, excluding *Wolbachia* (e.g., exposing the insects to the feces of non-treated individuals). *Wolbachia* does not appear to be efficiently transmitted via ingestion (e.g., Faria et al., 2016), but see discussion on horizontal transmission routes below. It is essential to monitor *Wolbachia* infection status of antibiotic-treated host strains, because antibiotics may not always fully remove infection. Instead, they may reduce *Wolbachia* densities to non-detectable levels in one or few generations (Li et al., 2014); this has been our experience in both *Anastrepha* (S.B. Lanzavecchia, C. Conte, and D.F. Segura, pers. obs.) and *Drosophila* (M. Mateos, pers. obs.).

Unidirectional CI is tested by comparing the embryo hatching rates of the CI cross (uninfected female X infected male) to that of one or more control crosses. For testing bidirectional CI, the reciprocal crosses of hosts infected by the different *Wolbachia* strains are assessed. A significantly lower embryo hatching rate of the CI cross(es) compared to that of the control cross(es) constitutes evidence of CI. CI can be partial or complete (100% embryo failure). As with any fitness assay, care must be taken to prevent potential biases, including crowding and age effects; which have been shown to influence CI (Hoffmann et al., 1990; Turelli and Hoffmann, 1995; Reynolds and Hoffmann, 2002). Adequate assessment of fertilization must be performed to ensure that failed embryos are not confused with unfertilized eggs. This may require testing for insemination of females that produce no larval progeny (e.g., Zabalou et al., 2009; Conte et al., 2019), or exclusion of females that predominantly lay unfertilized eggs, such as old *Drosophila melanogaster* virgin females (Menon et al., 2014).

## *Wolbachia* in Tephritids

### Taxonomic Distribution of *Wolbachia*-Tephritid Associations

Based mostly on PCR and sequencing approaches, ∼66% of ∼87 tephritid species screened have at least one record of positive *Wolbachia* infection (excluding pseudogenes) in laboratory and natural populations (see Supplementary File S1; only supergroups A and B have been found in tephritids). For the genus *Anastrepha*, all but one species (*Anastrepha ludens*) of 17 screened to date harbor *Wolbachia* (Werren et al., 1995; and this study; Selivon et al., 2002; Coscrato et al., 2009; Martínez et al., 2012; Mascarenhas et al., 2016; Morán-Aceves, 2016; Prezotto et al., 2017; Conte et al., 2019; Devescovi et al., 2019). Most *Anastrepha* species harbor *Wolbachia* strains assigned to supergroup A. *Anastrepha striata* Schiner and *Anastrepha serpentina* (Wiedemann), however, harbor supergroup B in southern Mexico (Martínez et al., 2012; and H. Martinez and M. Mateos, pers. obs.; see Supplementary File S1) and supergroup A in Brazil (Coscrato et al., 2009). Up to three *Wolbachia* sequence types have been detected per locality within morphotypes of the *A. fraterculus* complex (Prezotto et al., 2017; Conte et al., 2019), but co-infection of a single individual is generally not observed (except for one report in *A. fraterculus*; Cáceres et al., 2009).

Of the ∼49 species of *Bactrocera* that have been examined, ∼14 are reported to harbor *Wolbachia* (supergroup A and/or B) and three (*Bactrocera peninsularis* Drew & Hancock, *Bactrocera perkinsi* Drew & Hancock, and *Bactrocera nigrofemoralis* White & Tsuruta) carry what appear to be *Wolbachia*-derived pseudogenes, but not active infections (Kittayapong et al., 2000; Jamnongluk et al., 2002; Morrow et al., 2014, 2015). There is also the case of *Bactrocera zonata* Saunders, *B. dorsalis*, and *Bactrocera correcta* Bezzi that have been found to carry both active infections (cytoplasmic) and pseudogenized *Wolbachia* sequences (Asimakis et al., 2019). Up to five *Wolbachia* strains have been reported in a single individual of *Bactrocera ascita* Hardy (Jamnongluk et al., 2002), and double/multi infections have been reported in individuals of the following five *Bactrocera* species in Australia: *Bactrocera bryoniae* Tryon; *Bactrocera decurtans* May; *Bactrocera frauenfeldi* Schiner; *Bactrocera neohumeralis* Hardy; and *Bactrocera strigifinis* Walker (Morrow et al., 2014, 2015). Within the genus *Bactrocera*, polyphagous species are more likely to harbor *Wolbachia* compared to stenophagous or monophagous ones (Kittayapong et al., 2000).

For the genus *Ceratitis*, two species have been screened for *Wolbachia*. No evidence of *Wolbachia* was found in *Ceratitis fasciventris* Bezzi. Also, no evidence of infection was found in several populations of *C. capitata*, the Mediterranean fruit fly (medfly), in the early ‘1990s’ (Bourtzis et al., 1994). PCR amplification and sequencing of the 16S rRNA gene in several field and lab specimens of *C. capitata* from Brazil suggested infection with *Wolbachia* supergroup A (Rocha et al., 2005). However, recent thorough surveys of wild populations and lab colonies indicate that *Wolbachia* is absent in *C. capitata* from numerous localities in different continents (Supplementary File S1).

*Wolbachia* is reported in the four species of *Rhagoletis* examined to date: *Rhagoletis cerasi* L.; *Rhagoletis pomonella* Walsh; *Rhagoletis cingulata* Loew; and *Rhagoletis completa* Cresson (Zabalou et al., 2004b; Arthofer et al., 2009b; Drosopoulou et al., 2011; Schuler et al., 2011, 2012, 2013, 2016b; Augustinos et al., 2014; Bakovic et al., 2018). Both A and B supergroups are found in *R. cerasi* and *R. completa*, including a putative A–B recombinant strain, and co-infections are common (e.g., *R. cerasi* and *R. pomonella*).

In *Zeugodacus* (formerly *Bactrocera*), both *Z. cucurbitae* and *Z. diversa* are reported to harbor *Wolbachia* or *Wolbachia* pseudogenes (Kittayapong et al., 2000; Jamnongluk et al., 2002; Asimakis et al., 2019). Two out of the six species of *Dacus* examined to date are reported to harbor *Wolbachia*: *Dacus axanus* Hering (Morrow et al., 2014); and *Dacus destillatoria* Bezzi (Jamnongluk et al., 2002). *Wolbachia* has not been detected in the monotypic genus *Dirioxa* (Morrow et al., 2015). *Wolbachia* (supergroup A) has been reported in *Carpomya vesuviana* (Karimi and Darsouei, 2014) and *Neoceratitis asiatica* (Wang et al., 2019).

### *Wolbachia* Prevalence in Tephritids (in Time/Space)

Numerous studies report *Wolbachia* infection frequencies (or data from which this measure can be estimated) in natural populations of tephritids. Few of these studies, however, have adequate sample sizes for such inferences (e.g., many such studies are based on 10 or fewer individuals). Notwithstanding, inferred *Wolbachia* prevalence in tephritid populations is highly variable. In *Anastrepha*, ∼10 species harbor at least one population with prevalence ∼100%, whereas populations of three species reported lower frequencies (e.g., 88%, 51–60%, and 8.7%) (Supplementary File S1). In *Bactrocera*, one population of *B. caudata* had 100% prevalence, whereas all other species with positive *Wolbachia* results exhibited low prevalence.

The best studied tephritid system in terms of spatial and temporal variation in *Wolbachia* prevalence is that of *R. cerasi* in Europe, which was surveyed over a ∼15-year-period in 59 localities (Schuler et al., 2016b). Collectively, at least six strains *w*Cer1–6 have been identified from Europe and the Middle East. In an early (1998) survey, Riegler and Stauffer (2002) found all European *R. cerasi* individuals infected by one strain (*w*Cer1), most central and southern European populations harbored an additional strain *w*Cer2 (i.e., *w*Cer1 + *w*Cer2), and at least one Italian population harbored *w*Cer1 + *w*Cer4 (Zabalou et al., 2004b). A rapid spread of *w*Cer2 (a strain associated with cytoplasmic incompatibility) has been detected. Multiple infections, in various combinations of all five known *Wolbachia* strains from Europe, have been revealed recently. Samples from Poland, Italy, and Austria, are infected with strains *w*Cer1–5 those from Czech Republic (prior to 2009) and Portugal lacked *w*Cer2 only, while the Swiss samples lacked *w*Cer3 (Arthofer et al., 2009b). A more recent study of the Czech Republic (2015) and Hungary (2016) revealed that *w*Cer2 is spreading at a speed of 1.9 and 1 km/yr, respectively (Bakovic et al., 2018). Analysis of 15 Greek, two German and one Russian population confirm fixation for *w*Cer1 in all *R. cerasi* populations, and the presence of complex patterns of infections with four of the five known *w*Cer European strains (1, 2, 4, and 5) and the possible existence of new *Wolbachia* strains for the southernmost European *R. cerasi* population (i.e., Crete; Augustinos et al., 2014) and from Iran (*w*Cer6) (Karimi and Darsouei, 2014). Similarly, strain *w*Cin2 (which is identical to *w*Cer2 based on loci examined to date) is fixed in all populations of *R. cingulata*; a species native to the United States, but introduced into Europe at the end of the 20th century. Invasive populations in Europe harbor *w*Cin1 (identical to *w*Cer1 based on loci examined to date) at frequencies that vary over space and time (up to 61.5%), as a result of horizontal transfer (multiple events) from *R. cerasi* (Schuler et al., 2016b). The above studies indicate that the prevalence of *Wolbachia* types in *R. cerasi* and *R. cingulata* is highly dynamic.

### Phenotypic Effects of *Wolbachia* in Tephritids

Despite the numerous reports of *Wolbachia* in tephritids, the fitness consequences of such associations remain mostly unknown. The studies reporting phenotypic effects of *Wolbachia* have relied on transinfection and on antibiotic-curing; only two species of tephritids have been successfully transinfected with *Wolbachia* (Table 1). Evidence of *Wolbachia*-induced CI has been detected in four species of tephritids. Early studies (Boller and Bush, 1974; Boller et al., 1976) identified reproductive incompatibilities in *R. cerasi* that were later attributed to the *Wolbachia* strain *w*Cer2 (100% embryonic mortality in the CI cross; Riegler and Stauffer, 2002). Artificially transferred *Wolbachia* (strains *w*Cer2 and *w*Cer4) originally from *R. cerasi* to *C. capitata* also resulted in strong CI (100% embryonic mortality). *w*Cer2 in two genetic backgrounds of *B. oleae* resulted in strong CI as well (Apostolaki et al., 2011). In addition, *w*Cer2 and *w*Cer4 are bi-directionally incompatible in *C. capitata* (Zabalou et al., 2004b, 2009).

**TABLE 1:** Successful and unsuccessful *Wolbachia* transfection attempts in tephritids.

ID of successfully transfected tephritid strain	Donor species/strain	Recipient species (and strain)	*Wolbachia* strain	References
*C. capitata* WolMed 88.6	*R. cerasi*	*C. capitata* Benakeio strain	wCer2	Zabalou et al., 2004b
*C. capitata* WolMed S10.3	*R. cerasi*	*C. capitata* Benakeio strain	*w*Cer4	Zabalou et al., 2004b
*C. capitata* VIENNA 8-E88	*C. capitata* WolMed 88.6	*C. capitata* VIENNA 8 Genetic Sexing Strain (GSS)	wCer2	Zabalou et al., 2009
	*C. capitata* VIENNA 8-E88	*Bactrocera oleae*	wCer2	Apostolaki et al., 2011
N/A (unsuccessful)	*A. striata*	*A. ludens*	*w*AstriB	Martinez et al., 2016

In addition to CI, *Wolbachia*-infected *C. capitata* females (Benakeio strain) exhibit higher embryonic mortality (17–32% in crosses with *Wolbachia*-free males and 65–67% in crosses with *Wolbachia*-infected males) than their *Wolbachia*-free counterparts crossed with *Wolbachia*-free males (12% embryonic mortality). Therefore, it appears that *w*Cer2 and *w*Cer4 have additional fertility effects on medfly females, other than CI. It is also possible that these *Wolbachia* strains can only partially rescue the modification that they induce in sperm (Zabalou et al., 2004b). A similar pattern is reported in the Vienna 8 genetic sexing strain (GSS) infected with *w*Cer2 (Zabalou et al., 2009). The *w*Cer2 strain also causes increased embryo death in non-CI crosses of *B. oleae* (Apostolaki et al., 2011). In *D. simulans*, *w*Cer2 causes fecundity costs, moderate levels of CI, and incomplete rescue of its own CI modification (Riegler et al., 2004). Interestingly, a recent study examined the genome of *w*Cer2 and revealed the presence of three pairs of Type I *cif* genes and one Type IV *cifB* gene without a *cifA* complement, which might explain its idiosyncratic expression of CI (Morrow et al., 2020).

Two studies conducted several years apart (Sarakatsanou et al., 2011; Kyritsis, 2016; Kyritsis et al., 2019) examined the effects of a single *Wolbachia* strain (*w*Cer2) on fitness components of two *C. capitata* genotypes (i.e., Benakeio and Vienna 8 GSS laboratory lines), as well as the effects of two different *Wolbachia* strains (*w*Cer2 and *w*Cer4) on a single medfly genotype (Benakeio). The following general patterns emerged (exceptions noted): (a) *Wolbachia* causes higher egg-to-larva mortality; (b) *Wolbachia* causes higher egg-to-adult mortality (exception: Vienna 8 GSS + wCer2 in Sarakatsanou et al., 2011); (c) *Wolbachia* shortens egg-to-adult development time (exception: Benakeio + wCer2 in Kyritsis, 2016; Kyritsis et al., 2019). In addition, Sarakatsanou et al. (2011) found that *Wolbachia* shortens both male and female adult lifespan (exception: males of Vienna 8 GSS and *w*Cer2), and reduces life time female net fecundity. However, Kyritsis (2016) and Kyritsis et al. (2019) reported no effects of *Wolbachia* infection on adult lifespan, and a reduced fecundity in the case of *w*Cer4 infection only. Even though *w*Cer2 and *w*Cer4 in general tended to have consistent effects on medfly, the magnitude of their effects differed. Collectively, the results from these studies indicate that the effect of *Wolbachia* infection on life history traits depends both on the *C. capitata* genetic background and on the *Wolbachia* strain. Furthermore, inconsistencies between the two studies might be indicative of evolution of the host and/or *Wolbachia* strain during that period. Evidence of *Wolbachia* evolving reduced fitness costs has been reported in *D. simulans* (Weeks et al., 2007). Adult flight ability and longevity under stress conditions also appear to be determined by the interaction of *Wolbachia* strain and medfly genotype (Kyritsis, 2016; Kyritsis et al., 2019). A more recent study (Dionysopoulou et al., 2020) demonstrated *Wolbachia* effects on medfly reared in natural host fruits and at different temperatures. Medlfies infected with *w*Cer4 had low survival rates in both apples and bitter oranges, whereas those infected with *w*Cer2 were less vulnerable in apples than in bitter oranges. In addition, *w*Cer4 infected flies were particularly susceptible to high temperatures.

A recent study by Conte et al. (2019) examined the phenotypic effects induced by two *Wolbachia* strains native to *A. fraterculus* (sp1). No evidence of bidirectional cytoplasmic incompatibility was detected in reciprocal crosses among singly infected laboratory strains. However, the same work described the presence of slightly detrimental effects on larval survival and a female-biased sex ratio, suggesting the induction of male-killing phenomena. Moreover, Devescovi et al. (2019) found that *Wolbachia* reduced the embryo hatching in crosses involving cured females and infected males (uni-directional CI) within two morphotypes of this cryptic species complex; stronger CI was detected within the Peruvian morphotype than the Brazilian-1 morphotype (also referred as to “*A. fraterculus* sp. 1”). No evidence of bidirectional CI was detected in the crosses between the two morphotypes, leading Devescovi et al. (2019) to conclude that *Wolbachia* is not directly involved in the speciation process of these morphotypes. Ribeiro (2009) reported evidence consistent with CI caused by *Wolbachia* in *A. obliqua* and in *“A. fraterculus* sp. 1,” which according to *wsp* sequences, are identical. Nonetheless, confounding effects of the treatment to remove *Wolbachia* (removed by exposure of pupae to 30°C) or other potential biases cannot be ruled out, as all intraspecific crosses involving at least one cured parent resulted in much lower (<30%) embryo hatching than the intraspecific crosses involving both infected parents (66 and 81% embryo hatching).

Recent work demonstrates that *Wolbachia* infection can affect male sexual competitiveness of *C. capitata*. Different *Wolbachia* strains (*w*Cer2 and *w*Cer4) exerted differential impact on males mating competitiveness, and a single strain (*w*Cer2) had different impact on different medfly genotypes (Benakeio and Vienna 8 GSS laboratory lines) (Kyritsis, 2016; Kyritsis et al., 2019).

### Modes of Horizontal Transmission of *Wolbachia* Between Tephritid Hosts

Considering the dynamics of *Wolbachia* associated with arthropods in general, at the population level *Wolbachia* appears to be predominantly maintained by vertical transmission. Above the species level, however, the lack of congruence between the host and symbiont phylogenetic trees implies that *Wolbachia* horizontal transfers and extinctions are common and underlie its widespread taxonomic and geographic distribution (Bailly-Bechet et al., 2017).

The possible routes by which *Wolbachia* may be horizontally acquired by a new host can generally be classified as via ingestion or via a vector. In both cases, to become established as a stable cytoplasmically inherited infection, *Wolbachia* must cross one or more cell types or tissues. For example, if *Wolbachia* invaded the host hemolymph directly as a result of a vector (e.g., parasitoid wasp or ectoparasitic mite), it would have to invade the egg during oogenesis. Similarly, if *Wolbachia* were acquired via ingestion (e.g., as a result of scavenging), it would have to cross the gut into the hemolymph, before it invaded the egg. Support for the above routes comes from studies reporting: (a) that *Wolbachia* can retain viability outside cells and infect mosquito cell lines, as well as ovaries and testes that are maintained *ex vivo* (Rasgon et al., 2006; Hughes et al., 2012a); (b) that *Wolbachia* cells injected into *Drosophila* hemolymph reach the germline after crossing multiple somatic tissues (Frydman et al., 2006); (c) that *Wolbachia* can move between parasitic wasp larvae (*Trichogramma*) sharing the same host egg, and achieve vertical transmission (Huigens et al., 2004); and (d) that parasitic wasps of the white fly, *Bemisia tabaci* (Gennadius), can transfer *Wolbachia* from an infected to a naïve host, as a result of non-lethal probing (i.e., probing without oviposition), whereby the parasitoid ovipositor or mouthparts function as a “dirty needle” (Ahmed et al., 2015).

No direct evidence of *Wolbachia* transmission via parasitoids exists in tephritids, but sharing of *Wolbachia* strains between a parasitoid and several sympatric tephritids (Morrow et al., 2014; Mascarenhas et al., 2016) is consistent with parasitoid-mediated transmission, or transmission from tephritid host to parasitoid (Johannesen, 2017). The potential for horizontal transfer of *Wolbachia* among tephritids via parasitoids is high, due to the multiple instances where a single parasitoid utilizes several different tephritid host species (Quilici and Rousse, 2012; Murillo et al., 2016; Schuler et al., 2016a), and the high frequency of superparasitism by some fruit fly parasitoids (Tormos et al., 2012; Devescovi et al., 2017).

*Wolbachia* may invade a new host species via introgressive hybridization between two host species. This mechanism would also transfer mitochondria from the infected to the uninfected species nuclear background, akin to the artificial backcrossing approach described above (Figure 3). Ability of tephritids to hybridize in the lab has been reported in numerous species (Table 2), and hybridization in nature has been documented in *B. dorsalis/B. carambolae* (Wee and Tan, 2005), members of the *Ceratitis* FAR complex (Virgilio et al., 2013), and *R. cingulata/R. cerasi* in Europe (Johannesen et al., 2013). Thus, there is potential for wild tephritid populations to acquire *Wolbachia* infections via hybridization.

**TABLE 2:** Representative tephritid genera where hybridization between one or more species has been reported.

Tephritid genera containing species that can hybridize		Reference(s)
***Bactrocera***		
	***B. tryoni*** × ***B. neohumeralis***	Smith, 1979; Morrow et al., 2000; Meats et al., 2003; Pike et al., 2003
	***B. tryoni*** × *B. jarvisi*	Cruickshank et al., 2001
	*B. aquilonis* × ***B. tryoni***	Drew and Lambert, 1986
	*B. jarvisi* × ***B. neohumeralis***	Gilchrist et al., 2014
	***B. dorsalis^S^*** × *B. philippinensis****^S^***	Schutze et al., 2013
	*B. invadens****^S^*** × ***B. dorsalis^S^***	Bo et al., 2014
	***B. dorsalis^S^*** × *B. papayae****^S^***	Schutze et al., 2013
	*B. papayae****^S^*** × *B. philippinensis****^S^***	Schutze et al., 2013
	*B. papayae* × *B. carambolae*	Ebina and Ohto, 2006
	***B. tryoni*** × *B. jarvisi*	Shearman et al., 2010
****Ceratitis****		
	*C. rosa* × *C. fasciventris*	Erbout et al., 2008
****Anastrepha****		
	Within ***A. fraterculus*** complex	Selivon et al., 1999, 2005; Cáceres et al., 2009; Segura et al., 2011; Roriz et al., 2017; Rull et al., 2018
	***A. fraterculus*** × ***A. obliqua***	Dos Santos et al., 2001
	***A. sororcula*** × ***A. obliqua***	Dos Santos et al., 2001
	***A. fraterculus*** × ***A. sororcula***	Dos Santos et al., 2001
****Rhagoletis****		Schwarz and McPheron, 2007; Rull et al., 2010, 2012; Arcella et al., 2015; Tadeo et al., 2015
	*R. mendax* × ***R. pomonella***	Bierbaum and Bush, 1990; Schwarz and McPheron, 2007
	Within ***R. pomonella*** complex	Rull et al., 2010
	***R. completa*** × *R. zoqui*	Rull et al., 2012
	*R. pomonella* × *R. zephyria*	Arcella et al., 2015
	***R. cingulata*** × *R. indifferens*	Doellman et al., 2019
	Within ***R. cingulata***	Tadeo et al., 2015
	***R. cerasi*** × ***R. cingulata***	Johannesen et al., 2013
****Eurosta****		
	Within *Eurosta solidaginis*	Craig et al., 1997

*^S^B. papayae, B. philippinensis, and B. invadens are now considered junior synonyms of B. dorsalis (Drew and Romig, 2013; Schutze et al., 2015). Bold-face names are species where at least one report of Wolbachia infection exists (see Supplementary File S1).*

## Considerations for *Wolbachia*-Based Iit in Tephritids

**FIGURE 4:**
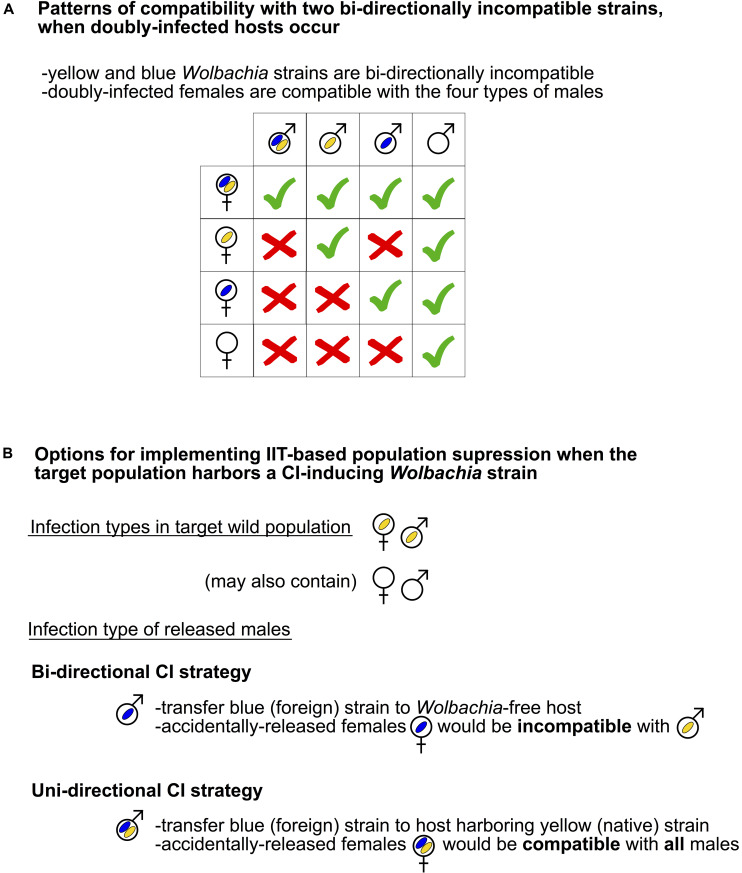
Use of bi-directional CI in IIT-based population suppression programs. **(A)** Patterns of compatibility with two bi-directionally incompatible strains, when doubly infected hosts occur. Empty male and female symbols signify absence of *Wolbachia*. Blue and yellow ovals represent distinct (mutually incompatible) *Wolbachia* strains. Green tick marks = Successful offspring production. Red crosses = no offspring production. **(B)** Options for implementing IIT-based population suppression when the target wild population harbors a CI inducing strain (yellow = “native”), according to the patterns of compatibility depicted in panel a. Bi-directional CI is achieved when the released males only harbor a strain (blue = “foreign”) that is incompatible with the native strain. Additional options exist, including double infections of both target and released insects with different *Wolbachia* strains (not shown), such as in *Aedes albopictus* (Moretti et al., 2018b).

There are two main approaches for implementing IIT, which depend on whether uni- or bi-directional CI will be used. If the target pest population lacks *Wolbachia*, such as the tephritids *C. capitata*, *B. oleae* (Gmelin), and *A. ludens* [and the mosquito *Aedes aegypti* (L.)], only unidirectional CI is feasible. In target populations that harbor one (or more) CI-inducing *Wolbachia* strain(s) (i.e., native strain; yellow in Figure 4), bi-directional CI can be achieved by releasing males that lack the native strain(s) and harbor one (or more) “foreign” *Wolbachia* strain(s) (blue in Figure 4) that is incompatible with the native strain. In contrast, if the released males are doubly infected with the native and foreign strains, the CI pattern employed for population suppresion is uni-directional (Figure 4).

In the case of Uni-CI patterns, the accidental release of *Wolbachia*-infected females, which would be reproductively compatible with wild and released males, may result in the replacement of the target pest population with a population harboring the *Wolbachia* infection of the released males, leading to failure of the IIT-based suppression program (Bourtzis, 2008). As described in Section “The Influence of *Wolbachia* on Host Ecology,” under certain conditions, a *Wolbachia* infection with frequency close to zero might be able to rapidly spread through a host population. Thus, without efficient sex separation mechanisms (outlined in section below), it is desirable to ensure that accidentally released females are sterile. In several tephritid systems, female sterility is achieved at a lower irradiation dose than male sterility, such as *A. ludens*, *A. obliqua*, *Anastrepha suspensa*, *A. serpentina*, *B. tryoni*, and *Z. curcubitae* (Toledo et al., 2004; Bakri et al., 2005; Rull et al., 2007; Collins et al., 2009). Therefore, an IIT program that used radiation at doses to ensure female sterility without compromising male quality (e.g., male competitiveness) could be effective (e.g., *Drosophila suzukii* based on results to date; Nikolouli et al., 2020).

In an IIT program based on bi-CI pattern (e.g., the recent field study of *Aedes albopictus*; Caputo et al., 2019), accidental release of fertile transinfected females, which would only be compatible with the released males, would not necessarily lead to population replacement and program failure. This is due to the generally higher threshold density required to achieve invasion (theoretically above 50% when the two incompatible *Wolbachia* strains exert equivalent fitness costs/benefits; see Section “The Influence of *Wolbachia* on Host Ecology”). Nonetheless, the actual outcome is strongly dependent in multiple factors (see Dobson et al., 2002; Moretti et al., 2018a). Therefore, for both uni-CI- and bi-CI-based IIT programs, as pointed out by Bourtzis et al. (2014), the outcome of accidental releases of infected females must be thoroughly evaluated via modeling and/or semi-field assays prior to field applications.

### The Advantage of Genetic Sexing Strains (GSS)

In general, SIT and IIT are most effective when only males are produced and released (Kerremans and Franz, 1995; Rendón et al., 2004). The release of only males in a large-scale operation can be accomplished by either killing female zygotes during development or by selectively removing them from the mass-reared population prior to release (Robinson, 2002a; Gilles et al., 2014; Lutrat et al., 2019). Genetic sexing strains (GSS) are those in which individuals can be separated by sex prior to the adult stage on the basis of a sex-linked phenotype (Franz, 2005). The earlier in development the females are removed, the most cost-effective the mass rearing operation will be, as investment in growth of females would be null or minimized. In most tephritids, male sex is determined by the presence of the maleness factor on the Y chromosome (Pane et al., 2002). GSS based on male-linked [e.g., Y chromosome – autosome (Y;A)] translocations have been developed in a few species to produce conditional female lethality (e.g., temperature sensitive lethality during embryonic development) or a visual sex marker (e.g., pupal color). Examples of tephritid species for which GSS are available include *C. capitata* (Franz, 2005), *A. ludens* (Zepeda-Cisneros et al., 2014), *Z. cucurbitae* (McInnis et al., 2004), *B. dorsalis* (Isasawin et al., 2012), and *B. carambolae* (Isasawin et al., 2014). Unfortunately, despite substantial efforts, GSS are still lacking for most tephritid pests. The recent development of CRISPR/Cas9-mediated mutagenesis in tephritids, however, might enable a faster development of tephritid GSS (Reid and O’Brochta, 2016; Choo et al., 2017; see reviews by Ogaugwu and Durvasula, 2017).

### Choice and Evaluation of *Wolbachia* Strains

The target population and the donor colony should be thoroughly screened for *Wolbachia*, ideally with the higher sensitivity methods described in Section “Methods to Assess *Wolbachia* Infection Status,” to detect low-titer and multi-strain infections. The *Wolbachia* strain selected should cause strong uni-CI with a *Wolbachia*-free, or strong bi-CI with *Wolbachia*-infected, target population. The selected *Wolbachia* strain should be artificially transferred to one or more lab colonies, representative of the genetic background of the target pest population. Most cases of successful establishment of stable transinfected insect lines have relied on embryonic microinjection (Hughes and Rasgon, 2014). Introgressive backcrossing might be feasible in scenarios where geographically isolated populations of the same target species harbor distinct *Wolbachia* strains (e.g., *A. striata* in Mexico vs. *A. striata* in Brazil; Supplementary File S1).

A thorough biological characterization of the artificial host-*Wolbachia* association should be conducted, as both host background and *Wolbachia* strain are important determinants of CI expression and other relevant fitness parameters (Bourtzis and Robinson, 2006; reviewed in Bourtzis, 2008; see also Kyritsis et al., 2019). The main desired characteristics of the association are: strong induction of CI; no rescue by *Wolbachia* strain(s) present in the target population; small or no fitness cost for parameters relevant to the program. These fitness parameters can be classified into those related to a cost-effective mass production (e.g., female fecundity including embryo hatching success) and those related to the success of released males (e.g., mating and sperm competitive ability, as well as dispersal/flight ability). Some host-*Wolbachia* combinations result in higher female fecundity, such as *D. simulans* after many generations (Weeks et al., 2007) and *Drosophila mauritiana* Tsacas and David (Fast et al., 2011). In contrast, other host-*Wolbachia* combinations result in lower fertility (e.g., low embryo success in *C. capitata* and *B. oleae*; Zabalou et al., 2004b, 2009; Apostolaki et al., 2011). *Wolbachia* could affect male mating success by influencing assortative mating; a phenomenon detected in some studies of *Drosophila* (e.g., Koukou et al., 2006; Miller et al., 2010), but not others (e.g., Champion de Crespigny and Wedell, 2007; Arbuthnott et al., 2016; Cooper et al., 2017). Such influence of *Wolbachia* on mating preferences was questioned (Sharon et al., 2010) on the basis of evidence that gut microbiota influence assortative mating in *Drosophila* (Sharon et al., 2010; Ringo et al., 2011; Arbuthnott et al., 2016), a finding that itself has been questioned recently (Leftwich et al., 2017, 2018). In addition, at least one case has been reported where sperm from *Wolbachia*-infected males was less competitive (Champion de Crespigny and Wedell, 2006). Similarly, *Wolbachia*-infected *D. simulans* produce fewer sperm (Snook et al., 2000). All of the above parameters should be evaluated under relevant conditions known to interact with *Wolbachia*, such as temperature and nutrition (reviewed in Bourtzis and Robinson, 2006; e.g., Serbus et al., 2015; Corbin et al., 2017; Ross et al., 2019a), interaction with other microorganisms (e.g., Hughes et al., 2014; Ye et al., 2017), as well as male age, paternal grandmother age, and mating status (e.g., Karr et al., 1998; Awrahman et al., 2014; Layton et al., 2019).

### Other Considerations

#### Species Recalcitrant to *Wolbachia*?

Certain species or clades appear to be “resistant” to *Wolbachia* infection, based on their lack of infection in nature and the failure to achieve stable transfections. The reasons are unknown, but could involve host and/or bacterial factors. For example, none of the members of the diverse *repleta* species group of *Drosophila*, comprised mostly of cactophilic flies (Markow and O’Grady, 2005), has ever been found to harbor *Wolbachia* (Mateos et al., 2006). Similarly, due to numerous failed transinfection attempts, and the lack of natural infection in wild *Anopheles* mosquitoes, this genus was regarded impervious to *Wolbachia* (reviewed in Hughes and Rasgon, 2014). This view has been challenged by the successful establishment of *Wolbachia*-transfected *Anopheles stephensi* Liston (Bian et al., 2013), and the recent discovery of a natural stable *Wolbachia* infection in *Anopheles coluzzii* Coetzee & Wilkerson (Shaw et al., 2016). Nonetheless, reports of *Wolbachia* in other species of *Anopheles* have been called into question (Sicard et al., 2019). The lack of natural infections and transinfection failure in *A. ludens* may reflect a general refractoriness to *Wolbachia*. Nonetheless, initial attempts to transinfect *C. capitata* also failed and transfection with *Wolbachia* was attained subsequently with different *Wolbachia* strains (Zabalou et al., 2004b). Hence, transinfection attempts with additional *Wolbachia* strains may result in successful and stable infection in *A. ludens* as well.

#### Potential for Target Populations to Become Resistant to Sterile Males

There are two ways in which a target population may become resistant to the effects of released *Wolbachia*-infected males. The first is endosymbiont-based, whereby the target population may acquire (e.g., via horizontal transmission) a *Wolbachia* strain that can rescue the modification (sterility) induced by the strain present in the released males. Generally, such acquisition of a *Wolbachia* strain during the relatively short lifespan of a release program seems unlikely. Nonetheless, knowledge on the *Wolbachia* infection status and strain identity of interacting species, such as other fruit flies sharing the same host plant and parasitoids, might aid in the selection of *Wolbachia* strains that are unlikely to be compatible with strains that can potentially be horizontally acquired by the target population. Permanent screening of wild flies from the target population could provide valuable information in order to foresee potential lack of effectiveness of the method. Laboratory experiments in which the conditions for horizontal transmission are favored (or even forced) might also help to determine the probability of such phenomena to occur in nature.

The second mechanism is host-based, whereby pre- or post-mating selection on wild females to avoid or reduce fertilization by incompatible sperm (reviewed by Wedell, 2013), acts on standing (or *de novo*) genetic variation. Evidence consistent with the influence of *Wolbachia* on premating mechanisms comes from the observation that females and males of *Drosophila paulistorum* Dobzhansky and Pavan exhibit assortative mating according to the *Wolbachia* strain they harbor (Miller et al., 2010; Schneider et al., 2019). In addition, treatment with antibiotic (which removed *Wolbachia*) decreases mate discrimination in *D. melanogaster* (Koukou et al., 2006). The evolution of resistance to mating with mass-reared males by wild females can be potentially minimized by frequently refreshing the genetic background of the mass-reared strain, with or without artificial selection (McInnis et al., 2002; Gilchrist et al., 2012; Zygouridis et al., 2014; Quintero-Fong et al., 2016; Sánchez-Rosario et al., 2017), which is a routine process in mass-rearing programs aimed at countering inbreeding and adaptation to mass rearing that is detrimental the success of released males (Robinson and Hendrichs, 2005). Nonetheless, if the basis for mate discrimination were solely determined by *Wolbachia* infection state (e.g., if females could distinguish *Wolbachia*-infected vs. *Wolbachia*-uninfected males solely on the basis of a *Wolbachia*-encoded factor), refreshing the fly genetic background of mass-reared strain is unlikely to slow down the evolution of resistance to released males in the target population.

Several lines of evidence are consistent with the influence of *Wolbachia* infection on post-mating mechanisms. The existence of genetic incompatibility is predicted to favor polyandry (multiple mating by females) as a female strategy to minimize the probability of her eggs being fertilized by sperm from incompatible males (Zeh and Zeh, 1996). Consistent with this prediction, uninfected *D. simulans* females remate sooner than *Wolbachia*-infected females (Champion de Crespigny et al., 2008). Furthermore, *Wolbachia* modifies the length of the spermathecal duct of females of the cricket *Allonemobius socius* Scudder (Marshall, 2007), which in turn may afford the female greater control on the outcome of sperm competition (e.g., *D. melanogaster*; Miller and Pitnick, 2002). Finally, the fact that host background can influence the CI phenotype (reviewed by Bourtzis and Robinson, 2006), suggests that target populations may have genetic variants that are more resistant to CI, which could increase in frequency as a result of the strong selection exerted by the massive release of *Wolbachia*-infected males.

#### Potential Alternative Ways of Implementing *Wolbachia*-Based Approaches

The recent identification of *Wolbachia* “CI genes” offers potential alternative ways of harnessing reproductive incompatibility in control of pest tephritids. First, to identify strains with the desired characteristics, at least ability to induce CI, a productive endeavor might be to search for CI loci in the genomes of candidate strains being considered for IIT, prior to artificial transfer efforts. A candidate *Wolbachia* strain that lacks CI loci homologs, or that contains CI loci homologs that are highly similar to (and thus potentially compatible with) strains present in target population, should be avoided. Secondly, it may be possible in the future to genetically engineer *Wolbachia* strains with the desired characteristics (e.g., one or more specific CI operons) for IIT programs, or to replace strains used previously in a control program, as a means of addressing resistance phenomena (Sullivan and O’Neill, 2017). Finally, a thorough understanding of the CI mechanism might enable the development of IIT based on *Wolbachia* transgenes, rather than *Wolbachia* infection. This might be particularly helpful in the control of species that are resistant to *Wolbachia* infection. Nonetheless, the release of such genetically modified insects might not be feasible due to regulatory hurdles and lack of public acceptance.

It has recently been shown that some *Wolbachia* strains can provide protection against major pathogens and parasites of insects, including RNA viruses and bacteria (Hedges et al., 2008; Teixeira et al., 2008; Ye et al., 2013; Martinez et al., 2014). It is very common for pathogens to appear in rearing facilities. Thus, if a *Wolbachia* strain could simultaneously cause strong CI and protect against one or more pathogens (e.g., RNA virus), this would have multiple benefits in an operational *Wolbachia*-based population suppression program. Furthermore, a *Wolbachia* strain that does not induce (strong) CI, but protects against pathogens might be desirable in a program that does not rely on CI (e.g., SIT) for population suppression. *Wolbachia*-mediated pathogen protection would enable high production and quality levels, thereby contributing to a cost-effective and sustainable insect pest management program.

#### Potential Influence of Other Symbionts

Multiple studies have revealed that although *Wolbachia* appears to be the dominant facultative heritable symbiont of arthropods, numerous other diverse bacteria (e.g., *Spiroplasma*, *Arsenophonus*, *Rickettsia*, and *Cardinium*) form such associations with insects, causing a diversity of reproductive and non-reproductive phenotypes (reviewed in Zchori-Fein and Bourtzis, 2011; Hurst and Frost, 2015; McLean et al., 2018). Despite the long-standing recognition that “*Wolbachia* do not walk alone” (Duron et al., 2008), many studies of *Wolbachia* fail to rule out the association of their study organism with other facultative heritable symbionts. Even intensely studied groups in terms of heritable symbionts, such as tsetse flies (genus *Glossina*), can yield surprises of bacterial associates (e.g., the recent discovery of *Spiroplasma* in two species of *Glossina*; Doudoumis et al., 2017). With few exceptions (Martínez et al., 2012; Augustinos et al., 2015; Asimakis et al., 2019; Conte et al., 2019; Devescovi et al., 2019), research on tephritid facultative heritable bacteria has not examined the possibility of players other than *Wolbachia*. Therefore, we urge that such research include screens for other symbionts, including viruses, protozoans, and fungi.

Tephritids are hosts to non-heritable bacteria, generally harbored in their gut (for recent reviews see Noman et al., 2019; Raza et al., 2020). Whether *Wolbachia* influences tephritid interactions with other microbes, has not been evaluated, but evidence for such interactions exists for other systems (reviewed in Brinker et al., 2019). For example, in *Drosophila neotestacea* Grimaldi, James, and Jaenike, the presence of *Wolbachia* promotes the abundance of *Spiroplasma*, and is positively correlated with abundance of Bacteroidales and Lactobacillales (Fromont et al., 2019). Similarly, *Wolbachia* influences the microbiome of *D. melanogaster* (Simhadri et al., 2017) and *Armadillidium vulgare* (Latreille) (Dittmer and Bouchon, 2018). It is therefore important to evaluate interactions between *Wolbachia* and the microbiome that influence negatively or positively aspects of mass-reared tephritids used in IIT or SIT.

## Conclusion

Given the widespread occurrence of *Wolbachia* in tephritids and its known fitness consequences in this group of dipterans and in other host taxa, *Wolbachia* is likely an influential component of tephritid ecology and evolution. Further exploration of *Wolbachia*-tephritid associations is expected to reveal a diversity of effects, including interactions with other microbial partners, as seen in more extensively studied systems such as *Drosophila* and mosquitoes. The recent exciting progress in understanding the basis of CI, and many other aspects of *Wolbachia* biology, should accelerate progress in the development of *Wolbachia*-based IIT for tephritid species, particularly with the aid of comparative *Wolbachia* genomics to identify potential CI patterns on the basis of CI gene composition. We consider that one of the major obstacles to effectively implementing IIT will be to avoid population replacement due to accidental release of *Wolbachia*-infected females. The threshold number of accidentally released females, which is generally much higher in systems that employ bidirectional-CI compared to unidirectional-CI, must be thoroughly investigated prior to any field implementation. Where an unacceptable risk of population replacement exists, we recommend that SIT be explored as a complementary strategy to support IIT.

## Author’s Note

An earlier version of manuscript has been released as a Pre-Print at https://www.biorxiv.org/content/10.1101/358333v1.

## Author Contributions

MM led the drafting. HM, PL, JT, BM-A, KG, SL, DS, CC, AA, GT, EA, VD, NP, and GK edited multiple drafts.

## Conflict of Interest

The authors declare that the research was conducted in the absence of any commercial or financial relationships that could be construed as a potential conflict of interest.

## Footnotes

1 https://pubmlst.org/wolbachia/

## Supplementary Material

FILE S1 Compilation of published reports of screenings of *Wolbachia* (and other heritable bacteria) in pest Tephritidae. In our counts of species, *A. fraterculus* morphotypes (Hernández-Ortiz et al., 2015) are regarded as separate species. Additional references not cited in main text but cited in this table (Drosopoulou et al., 2010; Karimi and Darsouei, 2014; Yong et al., 2017; Gichuhi et al., 2019; Schebeck et al., 2019; Wang et al., 2019).
